# Geomorphic and Ecological Disturbance and Recovery from Two Small Dams and Their Removal

**DOI:** 10.1371/journal.pone.0108091

**Published:** 2014-09-18

**Authors:** Desirée D. Tullos, Debra S. Finn, Cara Walter

**Affiliations:** 1 Water Resources Engineering, Oregon State University, Corvallis, Oregon, United States of America; 2 Department of Integrative Biology, Oregon State University, Corvallis, Oregon, United States of America; University of Delhi, India

## Abstract

Dams are known to impact river channels and ecosystems, both during their lifetime and in their decommissioning. In this study, we applied a before-after-control-impact design associated with two small dam removals to investigate abiotic and biotic recovery trajectories from both the elimination of the press disturbance associated with the presence of dams and the introduction of a pulse disturbance associated with removal of dams. The two case studies represent different geomorphic and ecological conditions that we expected to represent low and high sensitivities to the pulse disturbance of dam removal: the 4 m tall, gravel-filled Brownsville Dam on the wadeable Calapooia River and the 12.5 m tall, sand and gravel-filled Savage Rapids Dam on the largely non-wadeable Rogue River. We evaluated both geomorphic and ecological responses annually for two years post removal, and asked if functional traits of the macroinvertebrate assemblages provided more persistent signals of ecological disturbance than taxonomically defined assemblages over the period of study. Results indicate that: 1) the presence of the dams constituted a strong ecological press disturbance to the near-downstream reaches on both rivers, despite the fact that both rivers passed unregulated flow and sediment during the high flow season; 2) ecological recovery from this press disturbance occurred within the year following the restoration action of dam removal, whereas signals of geomorphic disturbance from the pulse of released sediment persisted two years post-removal, and 3) the strength of the press disturbance and the rapid ecological recovery were detected regardless of whether recovery was assessed by taxonomic or functional assemblages and for both case studies, in spite of their different geomorphic settings.

## Introduction

Dam removal is increasingly implemented to address aging infrastructure and river restoration [1]. However, most dams have accumulated decades of sediment behind them that can become a concern for natural resources managers due to the potential for downstream deposition following removal. The deposition associated with sediment released during and following dam removal has the potential to generate an ecologically significant disturbance [2], where a disturbance is broadly defined as a discrete event that falls outside a predictable range for an ecosystem [3]. Physical disturbances (e.g. flooding, landslides) are defined as impacts to geomorphic systems that modify bed forms and features as channels react, relax, and respond to the disturbance [4]. Ecologically, physical disturbances result in the death or displacement of resident organisms [5]–[6]. Recent literature has emphasized the basis of [7], the need for [8], and results from (see [9] for review) studies that investigate interactions between abiotic and biotic responses to physical disturbance in order to describe how the timing and intensity of habitat disturbance, controlled by spatial and temporal variability in geomorphic processes, play an essential role in structuring biological communities.

In the context of dam removal, the sediment pulse released with decommissioning a dam can be considered a discrete event that acts as both a geomorphic and an ecological disturbance [10], and the biotic and abiotic responses to that disturbance can vary depending on the sensitivity of individual reaches to disturbance [11]. From a geomorphic perspective, rivers that are sensitive to disturbance undergo rapid and large changes as a result of a) features that make the channel not resistant to change, b) inadequate complexity and connectivity, c) large magnitudes of change in input conditions, and d) inadequate energy to process the disturbance [12]. In practice, this means that the sensitivity of the physical system will vary with features of the sediment pulse, including the material size, released volume, and timing of sediment release [13]–[18], relative to the background dynamics and geomorphic processes of the river [16], [19]. Ecologically, the sensitivity to disturbance is similarly defined by the prevailing environmental variability and stability [20]. For example, stream-dwelling species that occupy naturally variable habitats are typically adapted to the local disturbance regime [21]. This variability in sensitivities to physical disturbances leads to a range of responses to dam removal, such that concerns regarding some sediment pulses in some systems are not warranted [22], whereas other dam removals clearly generate physically and ecologically significant changes to the system [23]. Despite this knowledge of general processes surrounding the disturbance of dam removal, it is not clear under what conditions the dam removal generates disturbances that are of adequate magnitude and duration to impact aquatic ecosystems, what the spatial and temporal extent of the disturbance from any dam removal will be, or what recovery trajectory a system may take following the disturbance of dam removal.

Both geomorphologic and ecological recovery are often stated as the key objective of river restoration projects (e.g. [24]–[25]), and yet recovery from dam removal is a complex interaction of impacts associated with the press disturbance (cf. [26]) imposed by the dam over its lifetime and the pulse disturbance of the removal of the dam. Recovery is defined in the field of geomorphology as the return to a prior landform [27], and in the field of ecology as the return to a prior ecological state [28]. Dam removal can facilitate recovery of pre-dam conditions by restoring the natural regime of material fluxes [1], but the disturbance associated with a pulse release of sediment can generate varied effects on habitat complexity [29]–[31] and direct ecological impacts (e.g. [30], [32]). Hence, the trajectory of geomorphic and ecological recovery is likely to be influenced both by removal of the press disturbance represented by the dam itself and by generating a pulse disturbance associated with the removal event.

As in other restoration actions, benthic macroinvertebrate assemblages are shown to be useful indicators of the ecological disturbance and recovery trajectory following dam removal, and there is accumulating evidence that negative effects of dam removal on benthos are transient (<1 year post dam removal [30], [33]–[37]). Existing studies have primarily relied on measures of change in the taxonomic structure of macroinvertebrate assemblages. However, individual taxa possess unique traits that may benefit them in the altered habitats immediately downstream of dams (e.g. mussels [38]) or in the unstable habitats (e.g. drifting and multivoltine invertebrates [39]) that can occur downstream of dam removals. Shifts in the dominance of such traits downstream of dam removals may provide more targeted insight regarding the longer-term effects of both the press and pulse disturbances associated with dams and their removal. Many macroinvertebrate taxa have been well characterized according to suites of functional traits that are expected to respond directly to habitat and disturbance filters (cf. [40]) associated with changing physical conditions [39], [41]–[43]. Disturbance effects on some of these functional groups, especially those with sensitivities to fine sediment deposition (e.g. a clinging habit, respiration with external gills), may persist well beyond the shorter-term effects assessed generally with taxonomic characterization of the community [32], [44].

In this study, we applied a before-after-control-impact (BACI) design associated with two small dam removals in an attempt to investigate recovery trajectories from both the press disturbance associated with the presence of dams and the pulse disturbance associated with removal of the dams. The two study sites occupy distinct physiographic settings that we expected to represent low and high sensitivities to the pulse disturbance of dam removal, given differences in background environmental variability and the relative size of the dams. We evaluated both geomorphic and ecological responses annually for one year prior to and two years following dam removal, and asked if functional traits of the macroinvertebrate assemblages could provide more persistent signals of ecological disturbance than taxonomically defined assemblages over the period of study.

## Materials and Methods

### Study sites

**Figure 1:**
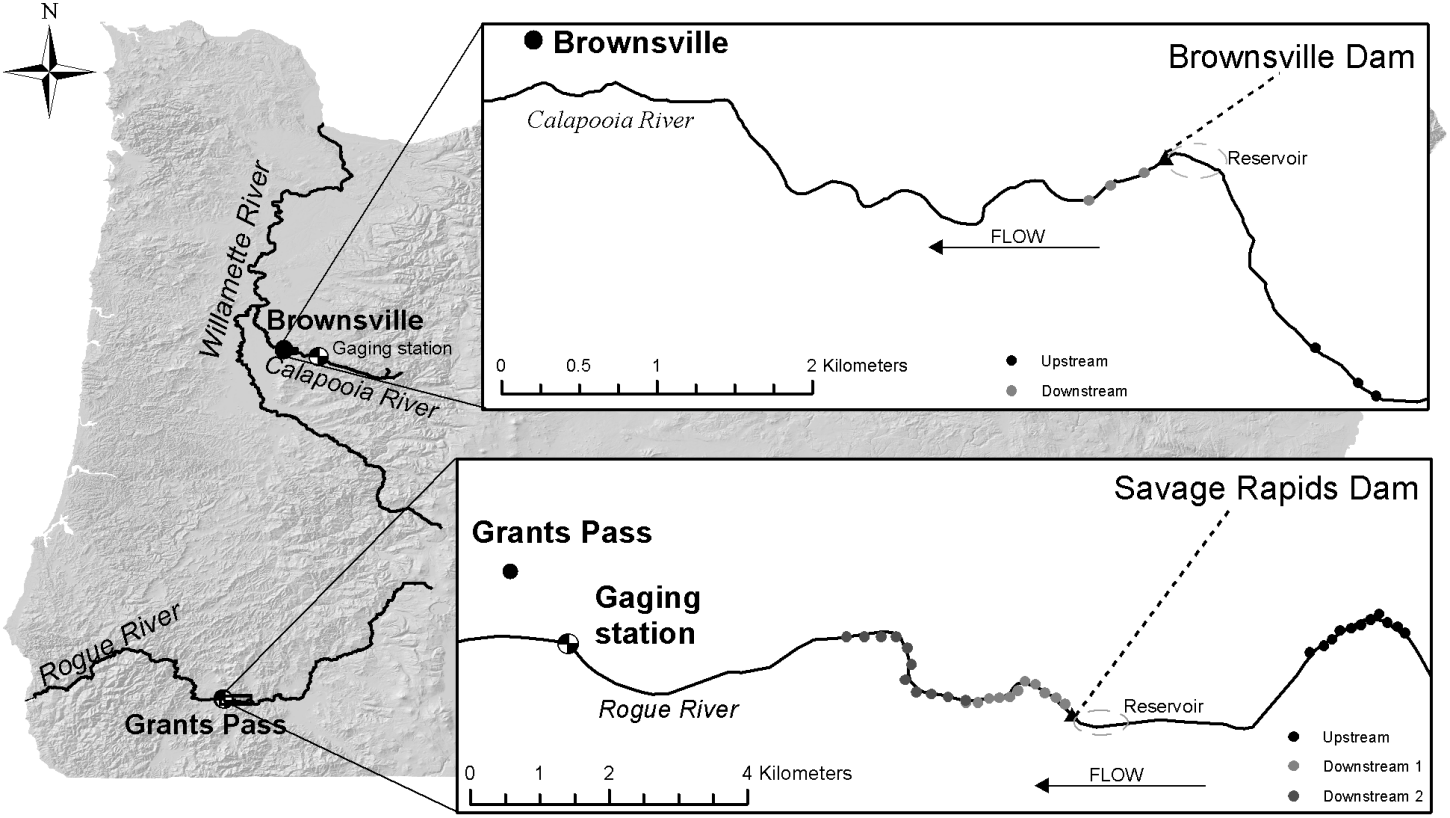
Site locations. Each dot represents a macroinvertebrate sampling location: 3 riffles per reach on the Calapooia River, and 11 transects per reach on the Rogue River.

The Calapooia River runs 121 km west from Tidbits Mountain in the Western Cascade Range to Albany, Oregon where it joins the Willamette River (Figure 1). Brownsville Dam (122°56′0.05″W 44°23′15.67″N) was a run of river, low-head dam (Table 1). The dam was originally built as a log crib dam in the late 1800s as a diversion and was rebuilt after failure in the 1964 flood [45]–[46]. The study area includes two reaches: 1) a 0.67 km control reach (C-US), a length equal to twenty times active channel width, that was located 2 km upstream from the dam; and 2) a 0.67 km (C-DS) impact reach starting immediately downstream of the dam (Figure 1). Permission for access was not necessary since work was conducted within the high-water elevation of the river.

**Table 1:** Characteristics of the study reaches and sediment reservoirs.

Dam name	Brownsville	Savage Rapids
River	Calapooia	Rogue
Drainage area above dam (km^2^)	404	6369
Location of dam (River km)	62	173
Dominant catchment land use	private forest and agriculture	public and private forest
Catchment mean annual precipitation (mm)	1730	1400
Dam function	mill and aesthetic diversion	irrigation diversion
Year removed	2007	2009
Reason for removal	fish passage and safety concerns	fish passage concerns
Barrier height (m)	2.4–4	9.1–12.5
Active channel width (m)	35	90
Avg. Width: Depth	34	21
Slope (m/m)	0.002	0.003
Stored sediment volume (m^3^)	17,000	543,000
Erosional efficiency	10^−5^	10^−4^
D50_R_/D50_D_	2.8	0.18
D50_R_ (m)	0.06	0.008

Barrier height varies with season due to installation of flashboards (Brownsville Dam) and stop logs (Savage Rapids Dam). Erosional efficiency was calculated as dimensionless ratio of the volume of sediment eroded from the reservoir to the volume of streamflow delivered to the site across the study period. D50_R_ = median grain size of the reservoir sediments. D50_D_ = median grain size of the river bed downstream of the dam.

The Rogue River flows 346 km west from springs on Mount McLoughlin in the Cascade Range near Crater Lake to the Pacific Ocean at Gold Beach, Oregon (Figure 1). Savage Rapids Dam (123°13′46.85″W 42°25′14.30″N) was built in 1921 as an irrigation diversion (Table 1) [47] during the summer, passing peaks flows during winter rainfall and spring snowmelt runoff [48] unregulated. Decommissioning of Savage Rapids Dam began with the removal of the right bays of the dam in April–June 2009 during which time there was a small release of sediment associated with drawdown and the partial failure of the coffer dam [49]. The coffer dam remained in place and flow was forced through the left bays and fish ladder until October–November 2009, when the coffer dam and remaining infrastructure was removed, a pilot channel was constructed, and the river was returned to a free flowing river [50]. The study area consisted of three reaches (Figure 1): 1) a 1.5 km control reach (R-US), approximately 40 times summer wetted width, located 4 km upstream from the dam; 2) one 1.8 km impact reach, starting immediately downstream of the dam (R-DS1); and 3) a second 2.4 km impact reach (R-DS2), starting immediately below R-DS1. Permission for access was not necessary since work was conducted within the high-water elevation of the river.

These two sites represent potential differences in the sensitivity to the pulse release of sediment with dam removal. For example, the Calapooia River downstream of Brownsville Dam was expected to be less sensitive to the sediment pulse associated with the dam removal than the Rogue River downstream of Savage Rapids Dam. Reasons due to a lower erosional efficiency (cf. [18]) and coarse material in the reservoir, relative to the bed downstream (Table 1), on the Calapooia River. In contrast, higher erosional efficiency and a relatively small grain size of material stored in the reservoir indicated that the Rogue River may be sensitive to the geomorphic disturbance associated with a released sediment pulse in the sense that it is not resistant to change and has adequate energy to process the disturbance. The higher sensitivity at Savage Rapids Dam should confer shorter response and relaxation times [12]. However, while the two sites likely possess differences in their sensitivity to the sediment pulse and vary in the size of the dams and rivers (Table 1), they both a) stored in reservoir the equivalent volume of approximately one to two years of sediment yield from the basin; and b) passed sediment through or over the dam and thus downstream reaches were not supply limited.

### Data collection

Summer field surveys were conducted prior to and following dam removal. On the Calapooia River, the pre-removal survey was conducted in July 2007, prior to the dam removal in September 2007. Post-removal surveys on the Calapooia River were conducted in August 2008 and July 2009. On the Rogue River, pre-removal surveys were conducted in September 2008. Surveys were also conducted in July 2009, a time between in-water work periods when some construction had occurred but the channel had not yet been returned to a free-flowing river. Post-removal samples on the Rogue River occurred in August 2010 and July 2011. Surveys included benthic macroinvertebrates sampling, bed material substrate characterization, and topographic surveys. As described below, survey methods varied between the two sites due to the sizes of the rivers at the study sites. The Calapooia River is primarily wadeable while the Rogue River is not wadeable. All data from this study have been published in a data library online at http://rivers.bee.oregonstate.edu/.

#### Hydrology

**Figure 2:**
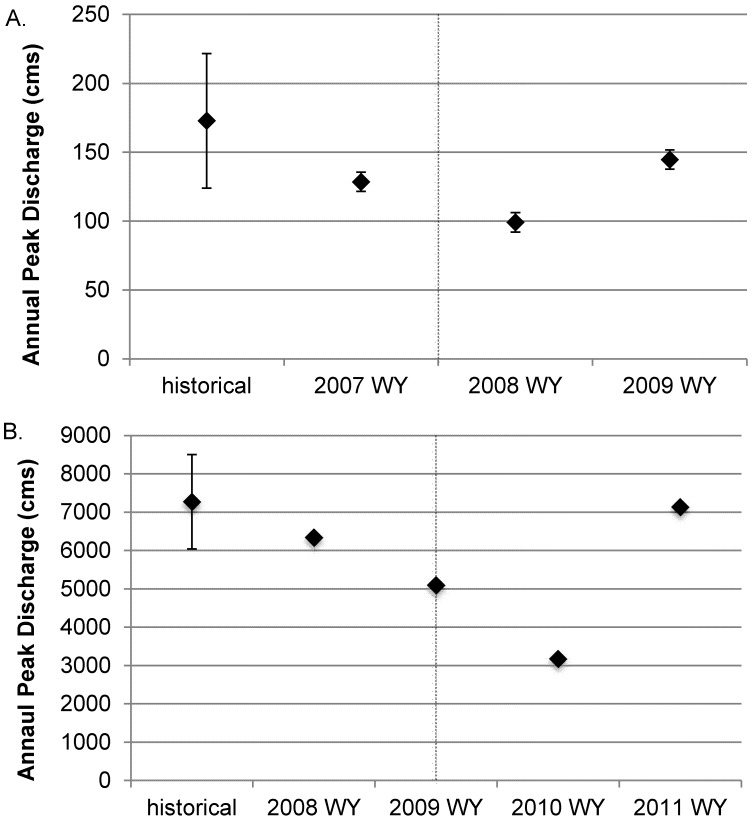
Annual peak discharge for the historical period of record and over the study period for the a) Calapooia at Holley, located 16 km upstream of Brownsville Dam, and b) Rogue River at Grants Pass located 8.6 km downstream of Savage Rapids Dam. Error bars on historical annual peaks represent 2 standard errors. Error bars for the Calapooia River for study years reflect estimation error associated with multiple regression estimate of peak discharge. Vertical dashed lined indicates approximate date of dam removal.

Despite growing evidence [18], [67] that the sequence of flows post dam removal has limited effect of the rates and styles of response to dam removal, we present the annual peak flow at both study sites for context. Hydrologic records were summarized as the annual peak discharge for the historical and study time periods (Figure 2). For the Calapooia River, there has not been a published record of discharge since the U.S. Geological Survey (USGS) stopped gaging the Calapooia River at Holley (#14172000), 16 km upstream of Brownsville Dam, in 1990. Therefore, we used two nearby USGS gages, South Santiam below Cascadia (#14185000) and Mohawk River at Springfield (#14165000), in basins of similar hydrogeology and land uses, to create a log-transformed regression model for annual peak flow for the Calapooia at Holley based upon 35 years of concurrent historical annual peaks [51]. The Rogue River is gauged by the USGS at Grants Pass (#14361500), 8.6 km downstream from the Savage Rapids Dam.

#### Geomorphology

On the Calapooia River, sediment samples were collected at two riffles per reach as 100-particle counts [52] for post removal years, as bulk samples [53] on two bars per reach in all years, and as bulk samples on two riffles per reach in pre-removal years [22]. Topographic surveys consisted of points taken with a total station (Nikon DTM 352) or real time kinetic (RTK) global positioning system (GPS) (Topcon Hiper Lite +) at slope breaks along four evenly-spaced cross sections per channel unit (riffle, glide, pool, run), along a longitudinal profile, and across bar surfaces [51].

On the Rogue River, water depth was measured and bed material was characterized by type (e.g. sand, gravel, bedrock) at 100–120 points on the thalweg along each study reach as part of Environmental Monitoring and Assessment Program (EMAP) surveys in 2008, 2009, 2010, and 2011. In addition, bankfull height above the low flow water surface was measured at eleven cross sections within each reach as part of the EMAP surveys [54]. More detailed bathymetry and water surface elevation were surveyed using a raft-mounted Acoustic Doppler Current Profiler (Workhorse Rio Grande) paired with a RTK GPS (Topcon GR-3). Surveys were conducted as one to three longitudinal profiles in 2010 and 2011, with variation in the number of profiles due to equipment failures and time constraints on field work. Pre-removal bathymetric surveys in the reservoir and downstream were performed by the U.S. Bureau of Reclamation (USBR) in 1999 and 2002.

#### Biotic communities

For the Calapooia River site, benthic macroinvertebrates were collected in riffles by disturbing sediment in front of a D-frame net for 60 seconds [55]. Samples were collected in three randomly selected locations per riffle at three riffles per reach on July 18, 2007, August 4, 2008, and July 14, 2009. On the Rogue River, samples were collected at the margin of each of eleven transects per reach using a D-frame net according to the EMAP non-wadeable protocol [54] on September 24–26 2008, July 29–30 2009, August 17–19 2010, and July 25–26 2011. All specimens were preserved in 70% ethanol.

### Characterization of geomorphic responses

#### Channel slope

For the Calapooia River, the mean channel slope for each reach was calculated as the average baseflow water surface slope based on edge of water elevations from the topographic surveys of cross sections for each year. For the Rogue River, channel slope was not measured in 2008 and 2009. Therefore, changes in slope on the Rogue River were not considered as part of this analysis.

#### D_gm_

For the Calapooia River, the geometric mean diameter (D_gm_) [56] for each reach was calculated as the average of the D_gm_ for all particle counts and surface bulk samples for that reach. For the Rogue River, type of substrate determined according to EMAP protocols [54] was converted to D_gm_ following the methods of [57] and averaged over each reach.

#### Variability of thalweg depth

For each of the sites, thalweg variability was calculated from thalweg profiles as a measure of habitat variability [58]–[59]. The standard deviation of thalweg depth was calculated for each study reach at each study site based on regularly-spaced thalweg depths. On the Calapooia River, thalweg depths were not directly measured. We calculated the thalweg depths as the vertical difference between a Triangulated Irregular Network (TIN) surface derived from the edge of water elevations for cross sections and bars, and the elevations of the longitudinal profile. As the longitudinal profile of the thalweg was measured at slope breaks and not regular intervals, we linearly interpolated the thalweg depth at a uniform spacing of 0.6 m from the thalweg depth derived from the longitudinal profile. On the Rogue River, thalweg depth was measured at constant intervals along each reach following the EMAP protocol [54].

**Relative Bed Stability.** We analyzed the stability of the riverbed using the modified relative bed stability (RBS*) index (Eqn 1) [60], a ratio between the mean particle diameter, D_gm,_ and the critical diameter at bankfull flow, D*_cbf_. The ratio is interpreted in relation to unity such that a ratio larger than 1 indicates the bed is likely stable during bankfull flows, whereas a ratio smaller than 1 indicates the bed is likely mobile during bankfull flows.
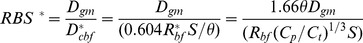
(1)where: D_gm_ = geometric mean bed surface particle diameter (m); D*_cbf_ = critical diameter (m) of bed surface particle at bankfull flow, averaged across the reach, and adjusted for shear stress reductions due to wood and depth variation; R_bf_ = bankfull hydraulic radius≈0.65d_th–bf_, where d_th–bf_ = mean thalweg depth + bankfull height above water surface (m); R*_bf_ = effective bankfull hydraulic radius (m) adjusted for wood and depth; S = energy slope, approximately the reach-scale water surface slope as a dimensionless ratio (m/m); θ = Shields number calculated from particle Reynolds number at bankfull flow {R_ep_ = [(gR_bf_S)0.5D_gm_]/ν}; C_p_ = stream reach-scale particle (grain) resistance at bankfull flow, calculated from reach wide mean relative submergence of D_gm_; and C_t_ = reach-scale hydraulic resistance at bankfull flow, calculated from bankfull thalweg mean depth and thalweg mean residual depth.

All parameters for calculating RBS* on the Rogue River were estimated based on the EMAP protocol [60], with residual depths calculated following a method consistent with [61]. On the Calapooia River, components for RBS* were calculated from the topographic surveys and analysis of sediment samples. For these assessments, bankfull height was estimated based on bank indicators observed in the field and verified as the highest elevation of depositional features in the Calapooia River. To estimate the residual depth on the Calapooia River, we calculated the elevation difference between each riffle crest and the thalweg in the pool immediately upstream of the crest, then averaged the residual pool depths across each reach.

### Characterization of biotic responses

We identified all macroinvertebrates to the family level for insects, amphipods, freshwater clams, and snails. Mites, oligochaetes, leeches, and flatworms were recorded at this coarser level (e.g. mites, oligochaetes, etc) of resolution. Our biological analyses relied on multivariate approaches applied to either taxonomically characterized assemblages for both insects and non-insects or on a functional characterization of only the insect component of the assemblages. We focused functional analysis on the insects because numerous functional traits are better understood for insects than for other benthic taxa. We assigned traits to each insect family following [39]. The suite of 20 traits, with a range of two to six trait modalities each (Table 1 in [39]), represents a variety of life history, mobility, morphological, and ecological functions that are expected to respond to varying aquatic habitat conditions (e.g. [62], [41]). Poff et al. [39] assigned trait modalities at the genus level, which we applied at the family level herein. In a few instances in which there was not a clear consensus modality for a particular trait across genera within families, we resolved the issue either by identifying our specimens to the genus level, when feasible, and assigning the appropriate trait modality or by choosing the most commonly occurring trait modality across the genera known to occur regionally within a family.

We used the software package PC-ORD [63] to run all multivariate analyses on the macroinvertebrate assemblages characterized both taxonomically and functionally. The fundamental sample units for these analyses, henceforth referred to as sites by year, are the samples from each year for each riffle (sum of three D-frame samples each) for the Calapooia River and for each EMAP transect (a single D-frame sample each) for the Rogue River (Figure 1). For each of the following multivariate analyses at the taxonomic level, we log(n+1) transformed raw abundance data to create the input matrices of *sites by year × taxon abundance*. We created input matrices for the multivariate functional analyses by multiplying matrices of *sites by year × raw taxon abundance* by a matrix of *taxa × trait modalities* for each of the twenty traits. The resulting matrices of *sites by year × absolute abundance of each trait modality* were then relativized, as the relative abundance of modalities within each trait at each site, to create final input matrices of *sites by year × relative abundance of each trait modality*.

We analyzed relationships of sites by year in multivariate ordination space. We used non-metric multidimensional scaling (NMS) to evaluate the influence of sample year and reach on the multivariate axis values of each assemblage. We used the multi-response permutation procedure (MRPP) in PC-ORD to assess whether there were differences in overall assemblage structure among sample years. MRPP results reported here include both an overall test for differences among groups, based on the significance of the statistic *A*, the chance-corrected within-group agreement, and tests for all possible pairwise comparisons between groups. We also used MRPP on single-year subsets of the two datasets to evaluate whether upstream and downstream reaches were significantly different in assemblage structure, either taxonomically or functionally defined, within any sample year. Significant differences between upstream control sites and downstream impacted sites following, but not prior to, dam removal would support a hypothesis of disturbance effects from the dam removal that persist up to or beyond a one year period, indicative of a year-plus recovery period. For each yearly subset, and for both rivers, we also conducted an indicator species analysis (ISA) [64] on the functionally-defined assemblage data in order to assess whether particular trait modalities were indicative of either upstream control or downstream reaches impacted by the dam and its removal. Under a hypothesis of downstream disturbance effects lasting beyond one year, we expected that trait modalities reflecting disturbance tolerance (e.g. multivoltinism, fast development, heavy body armoring, abundance in the drift) would be indicative of downstream reaches, while trait modalities reflecting intolerance to disturbed conditions (e.g. semivoltinism, slow development, respiration with gills) would be indicative of upstream reference reaches. For both the MRPP and ISA, we used a Bonferroni correction for multiple comparisons with alpha values equal to 0.05 to determine statistical significance.

In addition to the assessments of multivariate assemblage structure at the taxonomic and functional levels, we also quantified single metrics, one taxonomic and one functional, that are thought to be useful indicators of disturbance. We calculated percent of the total abundance of individuals that were Ephemeroptera, Plecoptera, or Trichoptera (%EPT), and percent abundance of the “clinger” modality of the habit trait (%clingers). Percent EPT is thought to respond negatively to disturbance and is a metric used frequently in bioassessment [65]–[66]. Although thus far not as widely used, %clingers describes the prevalence of a habit that requires abundant interstitial space in the substrate and a dominance of larger particle sizes, and clinger habit is expected to respond negatively to disturbance, particularly fine sediment deposition [44]. For each of these metrics, we used *t*-tests to assess differences between upstream and downstream reaches in each sample year and at both rivers. For these univariate analyses, we compared the reaches C-US and C-DS in the Calapooia, and the reaches R-US and R-DS1 in the Rogue. We excluded reach R-DS2 from this analysis because none of the prior multivariate analyses suggested any ecological response this far downstream of the former dam.

## Results

### Hydrologic context

On the Calapooia River, all three years of the study period experienced peak flows that were lower than the historical mean (Figure 2A). The first year post-removal was exceptionally low as it was less than two standard errors below the mean, whereas the pre-removal and second year post-removal experienced an annual peak flow that, while still low, were more typical of the historical peaks. The Rogue River experienced nearly the average annual peak flow in 2008, prior to dam removal, and an average annual peak flow in 2011, two years post-removal. Water year 2009, the year of the removal, experienced a peak flow of average magnitude whereas the lowest annual peak flow occurred in the water year following the dam removal.

### Geomorphic responses

Changes in physical habitat on the Calapooia River (Table 2A) indicate channel responses were associated both with the natural dynamism of the river and the removal of Brownsville dam. The downstream (C-DS) channel slope steepened in the first year post removal, without a similar change at the upstream reference site (C-US). In the second year post removal, the channel slope at C-DS flattened while the upstream site steepened. This pattern of steepening and flattening of the channel slope downstream of the dam removal is one indicator of the geomorphic disturbance and recovery associated with the pulse release of sediment.

**Table 2:** Geomorphic measures of disturbance from the sediment pulse.

A) Brownsville Dam on the Calapooia River					
Upstream (C-US)					
Year	Channel			Substrate	
	slope	Mean residual depth (m)	Std. Dev. of thalweg depth (m)	Dgm (mm)	RBS*
2007	0.0018	0.3	0.25	0.02	0.14
2008	0.0017	0.3	0.28	0.04	0.31
2009	0.0020	0.3	0.31	0.02	0.12
**Downstream (C-DS)**					
	**slope**	**Mean residual depth (m)**	**Std. Dev. of thalweg depth (m)**	**Dgm (mm)**	RBS*
2007	0.0028	0.3	0.32	0.01	0.13
2008	0.0033	0.2	0.27	0.04	0.28
2009	0.0025	0.3	0.25	0.02	0.22
**B) Savage Rapids Dam** **on the Rogue River**					
**Upstream (R-US)**					
**Year**	**Channel**			**Substrate**	
	**Mean residual depth (m)**		**Std. Dev. of thalweg depth (m)**	**Dgm (mm)**	RBS*
2008	0.9		0.9	0.04	1.75
*2009*	0.8		0.7	0.05	2.05
2010	0.9		0.7	0.05	1.65
2011	0.9		0.7	0.02	1.38
**Downstream (R-DS1)**					
	**Mean residual depth (m)**		**Std. Dev. of thalweg depth (m)**	**Dgm (mm)**	RBS*
2008	1.3		1.5	0.16	2.98
*2009*	1.4		1.4	0.05	0.79
2010	0.9		1.5	0.01	0.23
2011	0.4		0.5	0.01	0.18
**Downstream (R-DS2)**					
	**Mean residual depth (m)**		**Std. Dev. of thalweg depth (m)**	**Dgm (mm)**	RBS*
2008	1.3		2.5	0.24	6.16
*2009*	2.4		2.6	0.25	4.57
2010	2.9		2.8	0.01	0.27
2011	2.5		2.3	0.01	0.21

The year is underlined for sampling pre-removal for both sites, and italicized for sampling during the year of removal for the Rogue River.

**Figure 3:**
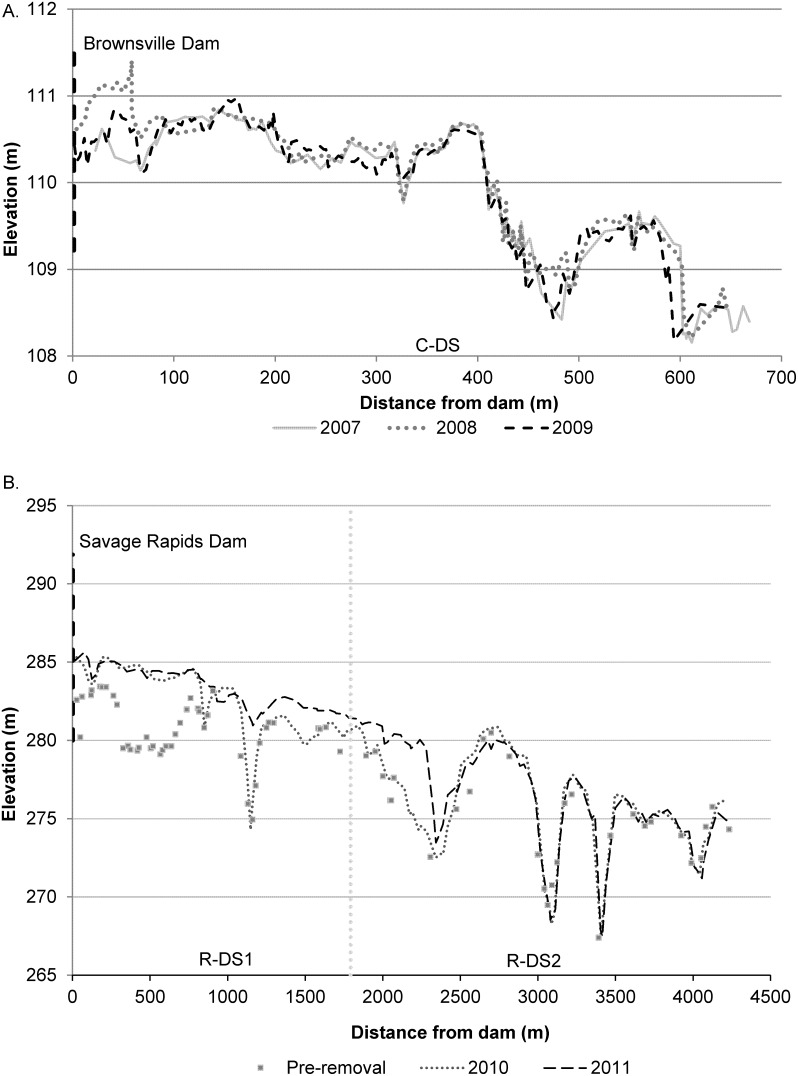
Longitudinal profiles downstream from the dams over time at a) Calapooia River and b) Rogue River. On the Rogue River, no longitudinal profile was collected during 2009, and the pre-removal profile is a compilation of surveys from 1999 in the farthest US and DS reaches and from 2002 for the main reservoir, both conducted by the US Bureau of Reclamation.

A second indicator of geomorphic response, bed variability, is represented by the standard deviation (SD) of the thalweg profile and the mean residual depth of the channel. On the Calapooia River, the thalweg SD increased in C-US while decreasing in C-DS between the pre-removal survey and the first year following removal (Table 2A). Also during the first year post-removal, mean residual depth did not change at C-US but decreased in C-DS as a result of sediment deposition in a pool immediately below the dam (Figure 3A). By 2009, the thalweg variability increased at C-US while C-DS underwent a further reduction in the thalweg SD. The downstream decrease in SD for 2009 was accompanied by a net increase in residual depth, associated with scouring of pools and deposition along some planar features in the channel. The anomalous, relative to the upstream control, decrease in variability, and reduction then increase in mean residual depth, downstream of the dam removal illustrates the effect of the sediment pulse on reducing thalweg relief in the Calapooia River.

**Figure 4:**
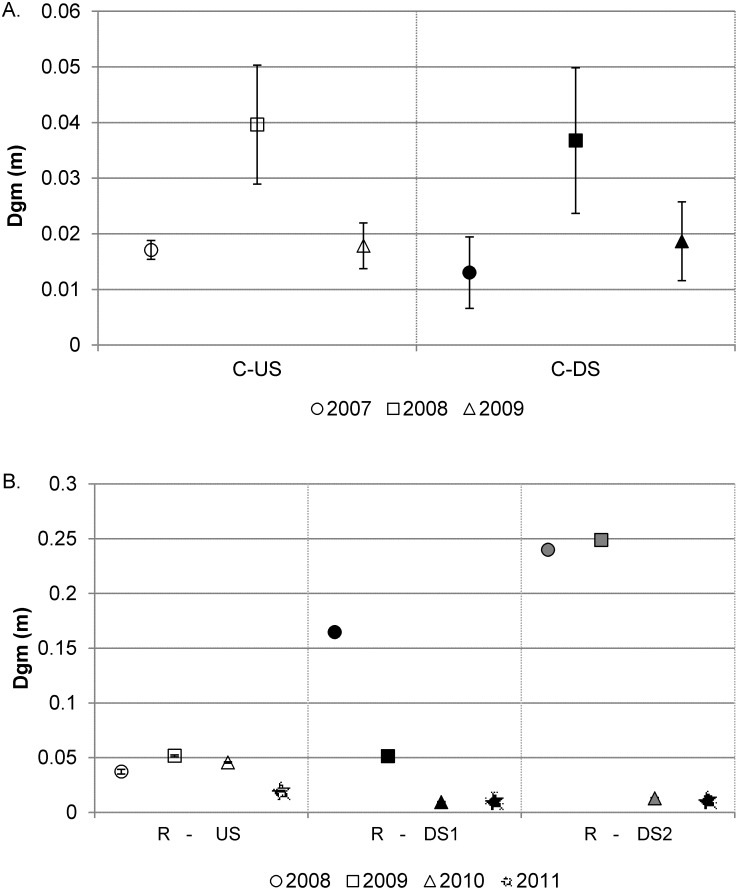
Plot of geometric mean grain size by reach over time at a) Calapooia River and b) Rogue River. Symbols represent the mean value, and lines represent two standard errors from the mean.

Finally, based on RBS* values (Table 2A), the bed in both C-US and C-DS was unstable before and after the dam removal and the reaches follow similar patterns of bed stability over time, though magnitudes of changes vary between the reaches. In the upstream reach, RBS* tracks changes in the dominant grain size (Figure 4A), increasing in the first year post-removal, then dropping in the second year post-removal. This trend indicates the basin underwent coarsening of the substrate during the lowest flows of the study in the 2008 water year (Figure 2A), resulting in higher stability of the bed, followed by a reduction in grain size and decreased bed stability during the more moderate flows of the 2009 water year. Similar trends were observed in both the grain size and RBS* for the C-DS, indicating that the sediment pulse had limited effect on the bed stability (Figure 4A) and that changes in bed mobility were more closely associated with the natural dynamism of the river than the dam removal.

On the Rogue River (Table 2B), we found evidence of geomorphic response to disturbance both with respect to bed variability and mobility. Variability of the bed was generally lowest at the upstream reference site for all years, and after a drop in 2009, remained constant over the study period. Likewise, mean residual depth varied little between years, indicative of a relatively stable channel. In R-DS1, thalweg variability exhibited little change during and for the first year following removal, though mean residual depth was reduced between 2009 and 2010. The reduction in residual depth reflects deposition in the deep pool, located ∼180–760 m downstream of the former dam. However, since no change in elevation was observed for the other two other pools in R-DS1 (Figure 3B), the range of depths did not appreciably adjust between 2009 and 2010 and thus the thalweg depth SD was steady. By 2011, the thalweg depth variability drops dramatically, accompanied by further reduction in the mean residual depth. These changes in bed variability were associated with deposition in the two pools located farther downstream in R-DS1 and aggradation on riffles located ∼1050–2350 m downstream of the dam. In R-DS2, the thalweg depth SD increased incrementally in 2009 and 2010, followed by a drop in variability in 2011. Mean residual depth followed a similar pattern of increase through 2010 followed by reduction in 2011. The increase in variability and residual depth through 2010 appears to be related to minor adjustments in the longitudinal profile independent of the dam removal whereas the reduction in variability and residual depth between 2010 and 2011 are associated with post-removal deposition in a pool located 1790–2170 m downstream of the dam. The time series of reduction in thalweg depth and variability in R-DS1 reflected the impact of pool filling immediately following removal, while the impact of the sediment pulse on relief was delayed in R-DS2 until two years post-removal.

Finally, RBS* indicates that the bed was generally stable at bankfull flows for all years in R-US and that dam removal reduced bed stability for the downstream reaches (Table 2B). For R-DS1, the bed was stable in 2008 but not in the year of or the years following dam removal. At R-DS2, the bed was stabile prior to and the year of dam removal, but not in the two years following removal. Bed stability in 2008 for R-DS1 and R-DS2, and 2009 for R-DS2 was primarily a function of the prevalence of boulders and bedrock (Figure 4B). However, the stability of the bed drops below one for R-DS1 in 2009, likely associated with the small amount of sediment that was flushed out of the reservoir with the spring 2009 drawdown of the reservoir for construction, which is evident as fining in R-DS1. In 2010, the first year post-removal, the grain size and RBS* values at R-DS1 were further reduced, and remained low in 2011, the second year post removal. R-DS2 also underwent substantial fining in 2010 and the grain size and bed stability remained low in 2011. Thus, while the grain size at the upstream control site varied little between years, the release of predominantly sand from behind the Savage Rapids Dam resulted in a reduction in dominant grain size downstream, with a concurrent reduction in bed stability due to the greater slope in R-DS1 and higher depths in R-DS2 relative to R-US.

### Biotic responses

In total, we identified 49 taxa, including 39 insect families, across all sites and years on the Calapooia River. We identified 38 taxa, including 27 insect families, across all sites and years on the Rogue River.

When characterized taxonomically, multivariate analysis of the full datasets revealed similar general patterns on the two rivers, including significant differences in assemblage structure among years, regardless of sample reach (Table 3). Both rivers also exhibited significant differences in taxonomic assemblage structure in upstream vs. downstream reach prior to initiation of dam removal activities, but no evidence of differences between reaches within any year following dam removal. The upstream-downstream differences on the Rogue River, when present, were only apparent in the pairwise comparison of reaches R-US vs. R-DS1. This pair of reaches was significantly different prior to the initiation of dam removal activities in 2008 and approached significance (p = 0.06) in 2009, the year during dam removal. Assemblages in reach R-DS2, over 1.7 km downstream of the former dam, were not different from either R-US or R-DS1 in any year.

**Table 3:** Results of MRPP analysis for differences among taxonomically defined assemblages in both rivers.

River	Dataset	Groups	Pairs	*A*	p
Calapooia	full	years		0.2	**<0.0001**
			07/08	0.2	**0.001**
			07/09	0.3	**0.0006**
			08/09	0.1	**0.001**
	2007	reaches		0.2	**0.02**
	2008	reaches		−0.05	0.7
	2009	reaches		0.01	0.4
Rogue	full	years		0.09	**<0.0001**
			08/*09*	0.1	**<0.0001**
			08/10	0.1	**<0.0001**
			08/11	0.07	**<0.0001**
			*09*/10	0.02	**0.006**
			*09*/11	0.03	**<0.0001**
			10/11	0.05	**<0.0001**
	2008	reaches		0.04	0.02
			US/DS1	0.06	**0.002**
			US/DS2	0.02	0.08
			DS1/DS2	0.003	0.5
	*2009*	reaches		0.02	0.06
			US/DS1	0.02	0.06
			US/DS2	0.02	0.1
			DS1/DS2	0.008	0.2
	2010	reaches		0.004	0.3
			US/DS1	0.002	0.4
			US/DS2	0.007	0.3
			DS1/DS2	0.002	0.4
	2011	reaches		0.02	0.09
			US/DS1	0.02	0.1
			US/DS2	0.02	0.1
			DS1/DS2	0.004	0.4

The first row of statistics for each group structure represents the test across the all groups comprised in that dataset, and the “pairs” column shows statistics for pairwise comparisons within the respective group. *A* is chance-corrected within-group agreement. Bolded p-values indicate significance at alpha = 0.05 after corrections for multiple comparisons.

For the functionally characterized insect assemblages (Table 4), patterns were broadly similar to those revealed for the taxonomically defined full macroinvertebrate assemblages. The functional assemblages in the Calapooia River were significantly different among all sample years in the full dataset, and the only significant differences between reaches C-US and C-DS within years was in 2007, prior to dam removal. On the Rogue River, functional assemblages also differ significantly among years in the full dataset, but the only significant pairwise difference was between 2008 and 2010, the year prior to and the first year following dam removal. Unlike the taxonomically defined assemblages, functional assemblages on the Rogue River did not differ significantly between any of the three reaches within any sample year.

**Table 4:** Results of MRPP analysis for differences among functionally-defined assemblages in both rivers, according to a suite of 20 life-history, mobility, morphological, and ecological traits.

River	Dataset	Groups	Pairs	A	p
Calapooia	full	years		0.5	**<0.0001**
			07/08	0.2	**0.002**
			07/09	0.5	**0.0005**
			08/09	0.5	**0.0007**
	2007	reaches		0.2	**0.03**
	2008	reaches		−0.03	0.8
	2009	reaches		0.03	0.4
Rogue	full	years		0.03	**0.003**
			08/*09*	0.02	0.02
			08/10	0.06	**0.0005**
			08/11	0.01	0.09
			*09*/10	0.005	0.2
			*09*/11	0.005	0.2
			10/11	0.02	0.04
	2008	reaches		0.007	0.3
			US/DS1	0.01	0.2
			US/DS2	0.03	0.1
			DS1/DS2	−0.03	1
	*2009*	reaches		0.03	0.1
			US/DS1	0.01	0.2
			US/DS2	0.02	0.2
			DS1/DS2	0.03	0.1
	2010	reaches		0.04	0.1
			US/DS1	0.03	0.1
			US/DS2	−0.002	0.4
			DS1/DS2	0.05	0.07
	2011	reaches		0.03	0.2
			US/DS1	0.05	0.09
			US/DS2	−0.03	1
			DS1/DS2	0.04	0.1

This table is organized and includes the same statistics as described for Table 3.

**Figure 5:**
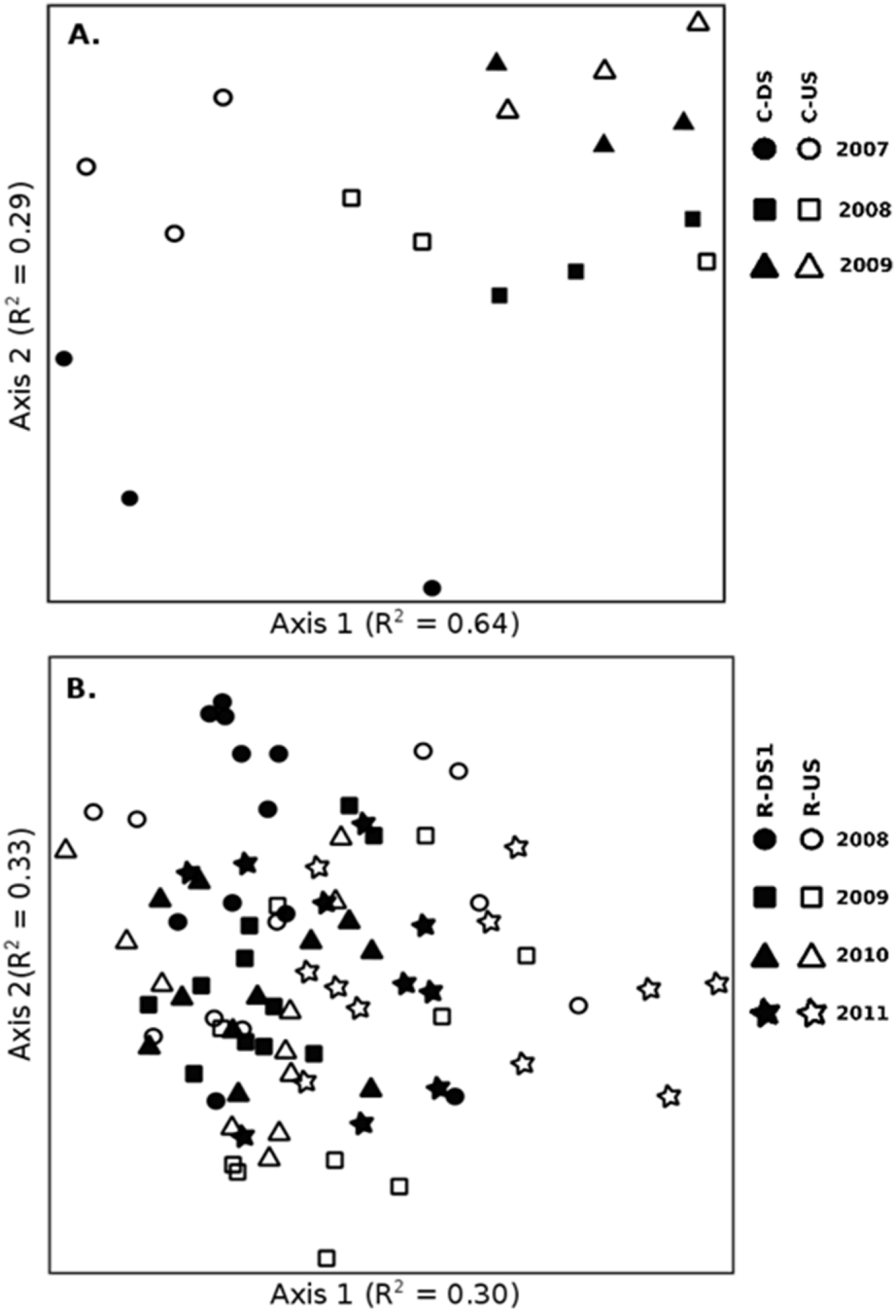
NMS ordination plot according to the taxonomic structure of A) 3 upstream and 3 downstream riffles associated with the Brownsville Dam removal on the Calapooia River over three collection years, and B) 11 upstream and 11 downstream transects associated with the Savage Rapids Dam removal on the Rogue River over four collection years. Note that the 11 transects from reach “DS2” have been removed from the figure to reduce clutter (see Table 3 for MRPP analysis of these data, including the DS2 reach). A) Final stress for the two-dimensional solution = 10.1. B) Final stress for a three-dimensional solution was 15.2, and the unpictured third axis had R^2^ = 0.21.

**Figure 6:**
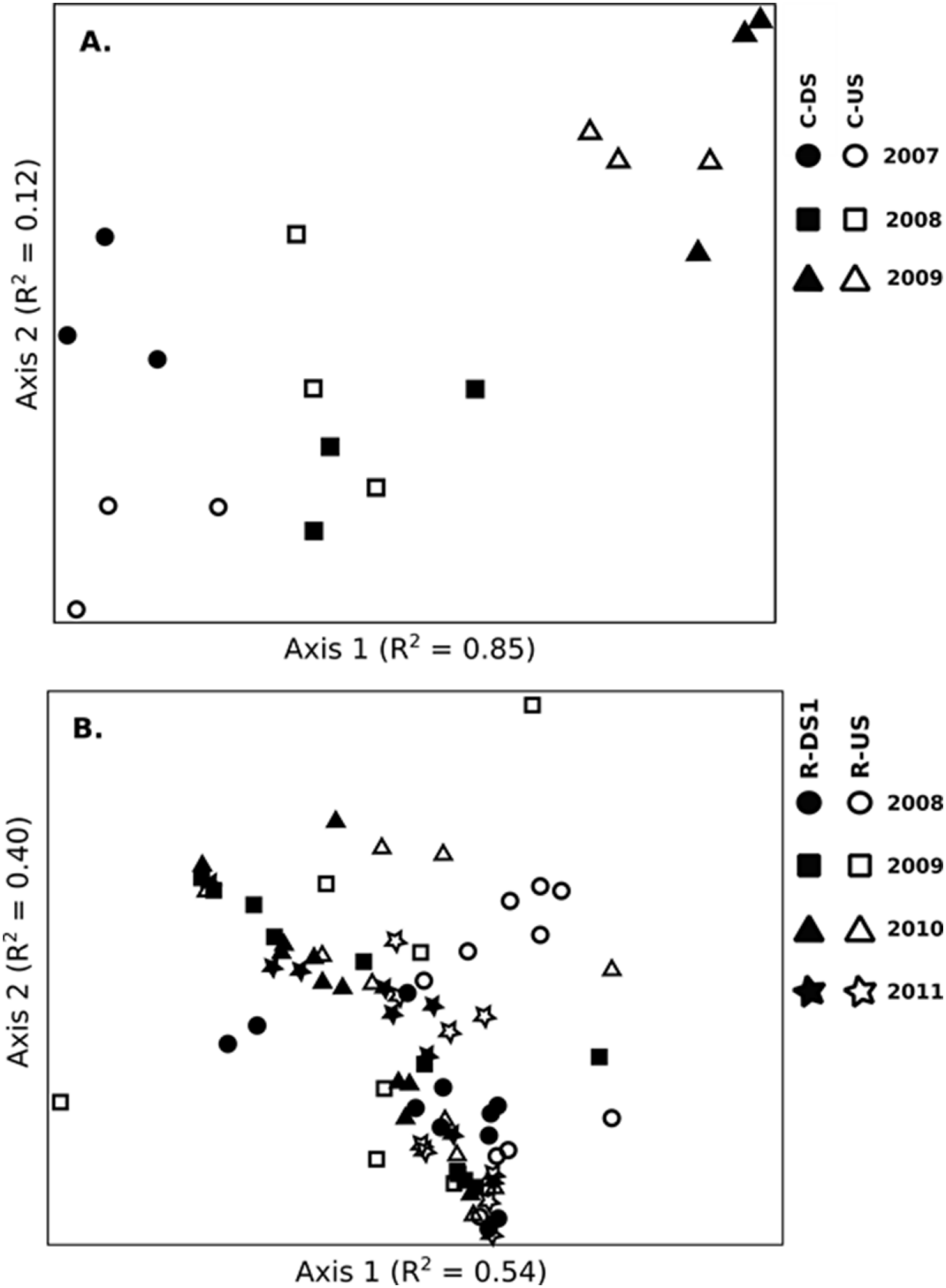
NMS ordination plot according to the functional community structure (based on 20 benthic insect traits) of A) 3 upstream and 3 downstream riffles associated with the Brownsville Dam removal on the Calapooia River over three collection years, and B) 11 upstream and 11 downstream transects associated with the Savage Rapids Dam removal on the Rogue River over four collection years. Note that the 11 transects from reach R-DS2 have been removed from the figure to reduce clutter (see Table 4 for MRPP analysis of these data, including the R-DS2 reach). The symbols follow the same explanation as in Figure 2. A) Final stress for the two-dimensional solution = 6.4. B) Final stress for the two-dimensional solution was 13.2.

To some extent, the differences observed in the pre- and post-removal assemblages were illustrated in the NMS plots. Taxonomic structure clearly delineated C-US and C-DS on the Calapooia River prior to dam removal along Axis 2, whereas the post-removal sites were not separated in ordination space (Figure 5A). Similar results are observed with ordinations of the functionally defined assemblages, with upstream and downstream sites on the Calapooia River clearly separated in ordination space prior to dam removal but not following dam removal (Figure 6A). In contrast, the pre-removal taxonomic distinction between R-US and R-DS1 is not evident on the Rogue River (Figure 5B), although upstream and downstream sites were separated to some extent along Axis 1 for the functionally defined assemblages (Figure 6B) prior to, but not following, dam removal.

Indicator species analysis on spatial distributions of trait modalities confirm the convergence of upstream and downstream sites post-removal, but also revealed somewhat different year-to-year patterns of upstream vs. downstream functional indicators between the two rivers (Table 5). We determined statistical significance at *α* = 0.05 following a Bonferroni correction, but “potential” indicators, those with an uncorrected p< = 0.05, were also listed in Table 5 to show trends. On the Calapooia River, the greatest abundance of functional indicators were in the year prior to dam removal (2007), in both reaches C-US and C-DS, followed by minimal, nonsignificant indicators distinguishing the two reaches in years following dam removal (Table 5A). Significant upstream indicators in the Calapooia River in 2007 were swimming habit, poorly synchronized emergence, and multivoltinism. Significant downstream indicators were well-synchronized emergence, univoltinism, and the herbivorous trophic group. In the same year, there were four other potential indicators of reach C-US, and three other such indicators at C-DS. At both one and two years post-dam-removal on the Calapooia, there were no significant indicators for either reach, although multivoltinism and a swimming habit scored uncorrected *p*-values of 0.04 and 0.05, respectively, as indicators of C-DS one year after dam removal. There were no significant indicators in either of the two post-removal years on the Calapooia River that clearly distinguished the upstream site.

**Table 5:** Indicator values (IV) and p-values from Indicator Species Analysis (ISA) comparing reaches for each trait modality with a *p*-value < = 0.05 for A) Calapooia River and B) Rogue River.

A) Calapooia	C-US			C-DS					
Year	Trait	IV	p	Trait	IV	p			
2007	poorly synchronized emergence	67.4	**0.0002**	well synchronized emergence	60	**0.0002**			
	multivoltine	89.3	**0.0002**	univoltine	59.5	**0.0002**			
	swim (habit)	87.8	**0.0002**	no swim ability	59.8	0.01			
	collector-gatherers	59.7	0.004	herbivores	63.1	**0.0004**			
	drift absent	61	0.01	clingers	53.6	0.004			
	weak swim ability	55.6	0.02	good armoring	74.5	0.03			
	high crawl rate	63.4	0.05						
2008	[none]			multivoltine	59.5	0.04			
				swim (habit)	58.9	0.05			
2009	[none]			high crawl rate	64.8	0.03			
**B) Rogue**	**R-US**			**R-DS1**			**R-DS2**		
**Year**	**Trait**	**IV**	**p**	**Trait**	**IV**	**p**	**Trait**	**IV**	**p**
2008	cool stenotherm	53.3	0.03	cold/warm eurytherm	34.4	0.002	[none]		
	rare in drift	55.4	0.005						
	high crawl rate	48.4	0.01						
	long-lived	47.3	0.05						
	semivoltine	38.1	0.05						
*2009*	univoltine	41	0.04	multivoltine	48.5	0.02	[none]		
	respiration with gills	36.2	0.03	poorly synchronized emergence	53.4	0.01			
	no armoring	36	0.05	swim (habit)	49.8	0.01			
	weak flight	35.9	0.02	streamlined shape	48.1	0.04			
2010	semivoltine	46	0.03	multivoltine	45.1	0.05			
	poor armoring	45.9	0.03						
	nonseasonal development	45.9	0.03				fast-seasonal development	36.1	0.008
2011	[none]			[none]			[none]		

Indicator trait modalities are organized into upstream and downstream reach columns by year-defined rows. Significant *p*-values following a correction for multiple comparisons are indicated in bold.

On the Rogue River, there were no significant indicators distinguishing the reaches for any of the years (Table 5B). However, in total, there were six potential indicator trait modalities distinguishing reaches R-US and R-DS1 in 2008, the year prior to dam removal, and eight potential indicator trait modalities in 2009, the year during removal. R-DS2 had no potential indicators in either of these years. The 2009 analysis suggested that multivoltine insects that do not use gills for respiration, have some degree of armoring, good swimming ability, and poorly synchronized emergence were found in greater abundance in R-DS1 relative to the R-US and R-DS2 reaches in the year that the dam was being removed. In 2010, the year following full dam removal, there were potential indicators of semivoltine insects with non-seasonal development and poor armoring in R-US in contrast to the single potential indicators in R-DS1, multivoltinism, and R-DS2, fast-seasonal development. However, by two years following dam removal, there were no potential indicator trait modalities distinguishing any of the Rogue River sample reaches.

**Figure 7:**
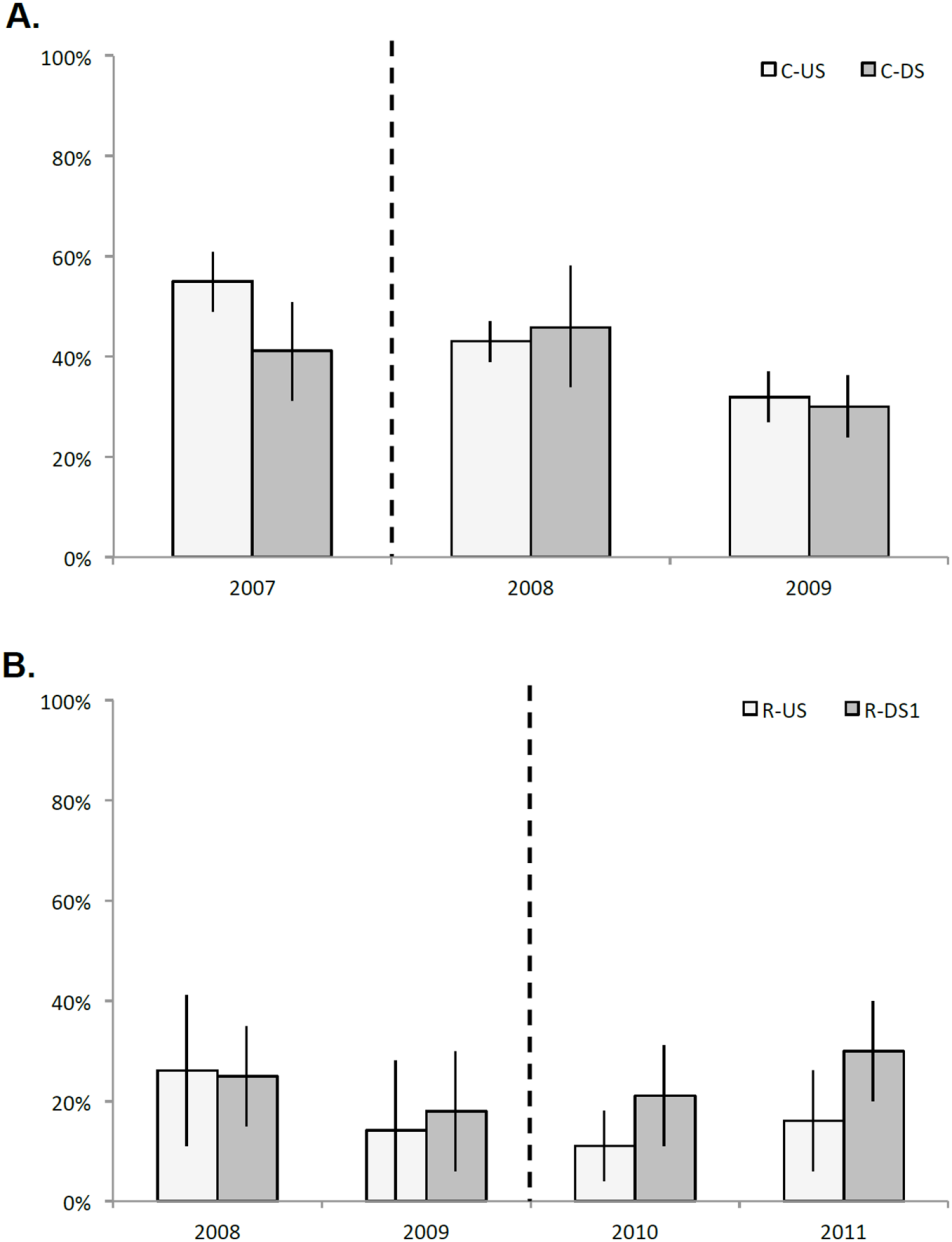
Mean %clingers (expressed as relative abundance, +/−2 standard errors) per reach across A) riffle sample units on the Calapooia, and B) transects sampled on the Rogue. In both panels, year is on the x-axis, and lighter-colored bars represent the upstream reaches. Vertical dashed line shows timing of dam removal. Note that, in the interest of space, the second downstream reach is not plotted for the Rogue due to the lack of change over time.

**Figure 8:**
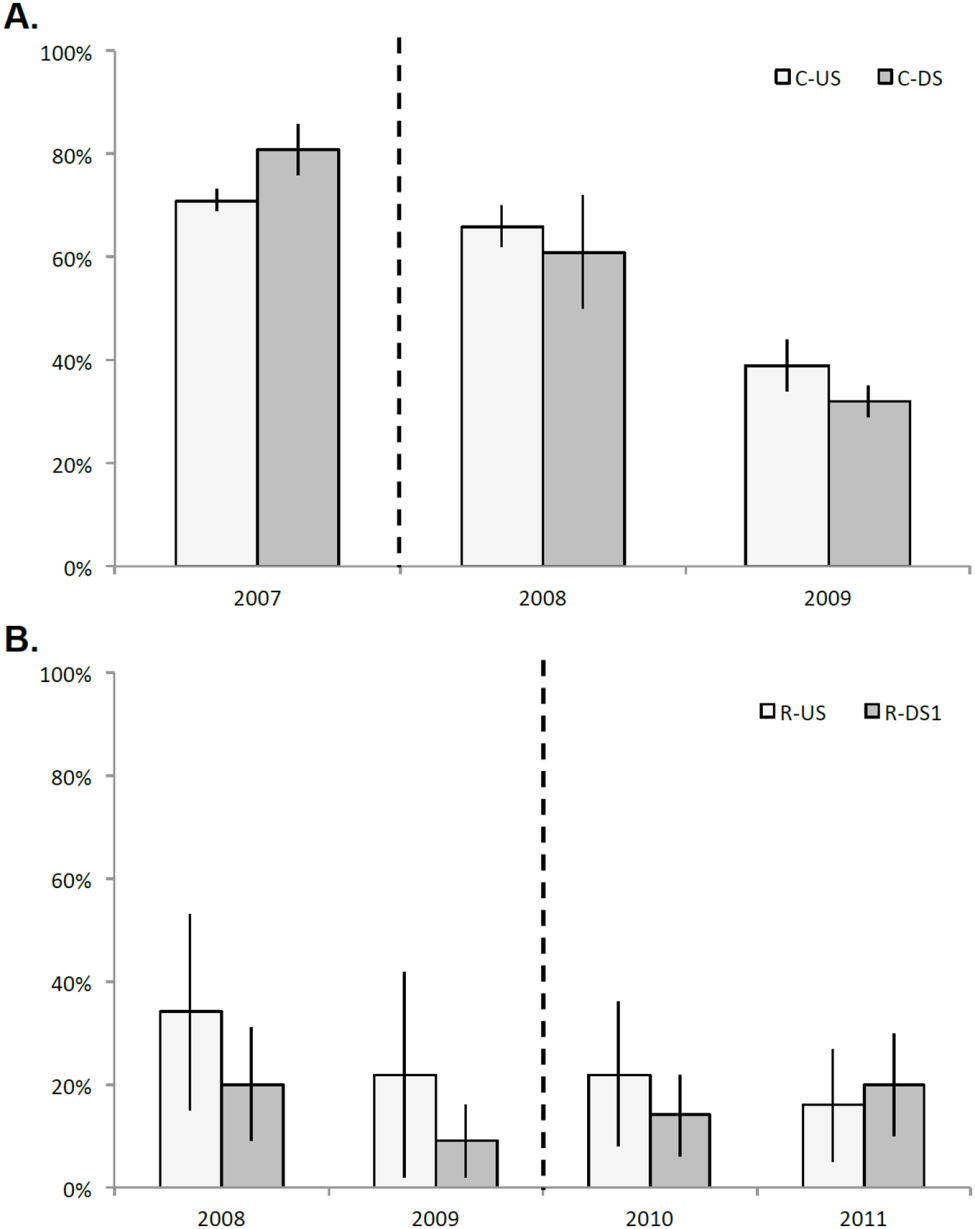
Mean %EPT (expressed as a proportion, +/−2 standard errors) per reach across A) riffle sample units on the Calapooia, and B) transects sampled on the Rogue. In both panels, year is on the x-axis, and lighter-colored bars represent the upstream reaches. Vertical dashed line shows timing of dam removal. Note that the second downstream reach is not plotted for the Rogue River due to the lack of change over time.

The univariate indicators %clingers (Figure 7) and %EPT (Figure 8) illustrate upstream-downstream trends across years, particularly in the Calapooia River, although only one of the within-year *t*-tests produced significant differences. With no statistical difference in % EPT across the years on the Calapooia River (Figure 8A), the only statistically significant difference on the Calapooia River was greater %clingers in C-DS than C-US in 2007, the year prior to dam removal (Figure 7A). In the two years following dam removal, the two reaches on the Calapooia River became statistically indistinguishable for %clingers. Values of %EPT were greater, though nonsignificant (*p* = 0.11), in C-US relative to C-DS for the Calapooia River prior to dam removal, followed by convergence of these reaches in the two years following dam removal. Year-to-year trends were difficult to assess and were nonsignificant on the Rogue River due to high reach-scale variability for both %EPT and %clingers (Figures 7B, 8B).

## Discussion

Dams are known to generate geomorphic and ecological impacts, considered to be press disturbances [68]–[69], but it is also possible [2] that the act of decommissioning these dams results in a pulse disturbance, both ecologically [24] and geomorphically [70]. We aimed to investigate recovery from both press and pulse disturbances in two physiographic settings with different dam characteristics, and to assess whether geomorphic and ecological recovery was consistent and concurrent. Overall, our results suggest that 1) the signature of geomorphic disturbance varied between the sites reflective of the physiographic setting and sensitivity of the rivers to the introduced sediment pulse, 2) the geomorphic disturbance from the sediment pulse persisted two years post-removal but ecological recovery of the macroinvertebrate assemblages, whether characterized taxonomically or functionally, occurred within a single year, 3) the signal of ecological recovery from removing the press disturbance of the dams is stronger than the signal of the pulse disturbance introduced by the removal of the dams, and 4) defining assemblages according to traits confirmed the impact of the press and pulse disturbances, but did not provide a stronger indication of lingering ecological disturbance than taxonomically-defined assemblages in our two systems.

From the geomorphic perspective, two physical signatures of the recovery trajectory emerged from the sites that are associated with the local geomorphology and characteristics of the sediment pulse. On the Calapooia River, we found evidence of disturbance and recovery based on 1) an initial increase then subsequent recovery of channel slope which was unmatched in the upstream control reach, and 2) a decrease in bed variability and residual depth that initially reduced bed relief. The release of coarse material to the reach downstream of Brownsville Dam had limited impact on the dominant grain size or bed stability, a predictable result given the small size of the dam and the transport of sediment over the dam for several decades. In contrast to the Calapooia River, the channel response to the sediment pulse on the Rogue River was generally reflected in a reduction in thalweg variability and residual depth, with impacts to the farther downstream reach delayed until two years following removal. In addition, the release of sand to the downstream reaches resulted in a substantial reduction in grain size and bed stability. Thus, the sediment pulse at both sites resulted in reduction in bed relief. However, the pulse of coarse sediment released to the Calapooia River was large enough to locally impact the channel slope but not bed material size and mobility, whereas the sand released to the Rogue River reduced bed material size and mobility.

While both sites exhibited some evidence of pulse disturbance for the two years following dam removal, we found limited evidence of the impact of the dam removals on the benthic macroinvertebrate assemblages. Instead, benthic assemblages appeared to rapidly respond to the removal of the press disturbance of the dam, rather than the introduction of a pulse disturbance associated with the release of sediment following removal. We inferred the ecological press disturbance of dams on both rivers through detection of significant differences in assemblage structure between upstream and downstream reaches in the years prior to dam removal. These upstream/downstream differences were detectable both taxonomically and functionally only in the year prior to dam removal. Downstream assemblages moved rapidly towards similarity with the upstream assemblages post-removal, as illustrated by the lack of significant differences between the upstream and downstream sites within a single year post-removal. This pattern suggests that ecological recovery occurred within a single year once dam removal activities had ceased, regardless of geomorphological differences between the two rivers. This rate of recovery is consistent with other studies [30], [33]–[37] that have documented annual-scale recovery from dam removal sediment pulses. In addition to the shorter timescale for ecological recovery, we found some evidence that the spatial extent for ecological disturbance is also smaller than for geomorphic disturbance. Reach R-DS2, the second downstream reach on the Rogue River, did not reflect any effects of press or pulse disturbance ecologically, but did reflect the pulse disturbance geomorphically in 2010 and 2011, suggesting that the spatial scale of the dam-removal disturbance was larger for the abiotic system than our measure of the biotic system.

We expected that, because individual trait modalities are often linked tightly to disturbance or other environmental filters, functional analysis would provide additional insight into the biotic effects of dam removal [44] over taxonomic approaches. As reflected in the MRPP, ISA, and ordination plots, the results of both the taxonomic and functional trait analyses clearly highlight that the upstream and downstream sites were significantly different prior to dam removal and converged towards similarity post-removal. However, taxonomic differences were stronger than functional differences on the Rogue River, and investigation of the individual indicator traits does not produce a clear interpretation of the disturbance of the dam or its removal. At the Calapooia River, pre-removal trait modalities associated with the upstream reach included both those that tend to be associated with disturbance (e.g. multivoltinism) and those associated with stability (e.g. poorly synchronized emergence, absence from the drift, high crawling rate). Similarly, the trait modalities at the downstream reach on the Calapooia River included a mix of traits that reflect both habitat stability (e.g. univoltinism, well-synchronized emergence, and the clinger habit) and disturbance (e.g. good armoring). Post-removal on the Calapooia River, the lack of distinguishing traits upstream and the decreasing number of disturbance-related traits downstream is indicative of converging functional modalities post removal. Across all years on the Rogue River, the benthic assemblages in the upstream reach were dominated by trait modalities representative of stable habitats, including indicators of long larval life span, high crawl rate, no or poor armoring, and rarity in the drift. In contrast, downstream sites did not strongly associate with any functional modalities prior to dam removal, except eurythermal taxa. During the year of dam removal, the reach immediately below the dam was dominated by traits associated with disturbance. We attribute this shift in the downstream community to dam decommissioning activities, including the potential transport of small volumes of fine sediment from the reservoir during the spring 2009 drawdown and subsequent construction. Only one disturbance-related trait persisted in the two reaches below the dam in the first year post-removal, and the assemblages had no distinguishing trait modalities two years post-removal. Thus, a mix of traits associated with the dams and their removal appear to characterize the communities in the year post-removal, but the dominance of any traits was eliminated at both sites by the second year following removal.

We anticipated that both clinger habit and %EPT would provide a mechanistic link between geomorphic and ecological responses to dam removal. Both respond negatively to disturbance, particularly deposition of sediments [32], [44]. However, our results indicate that these univariate measures alone were not strong indicators of the sediment pulse, largely due to the high variability in the assemblages. The only significant difference in %clingers upstream vs downstream of a dam was observed on the Calapooia River prior to dam removal, where the downstream impact reach had higher % clinger taxa than the upstream control site, potentially associated with the clay hardpan substrate downstream prior to removal. There was no difference in percent clingers between upstream and downstream reaches on the Rogue River regardless of year. Clingers also did not emerge as a potential indicator trait on the Rogue River, but did on the Calapooia River and only for the clay hardpan bed in the pre-removal downstream reach C-DS. The lack of differentiation between reaches using the %clinger metric, despite evidence from the physical data that the channels were unstable, suggests that the metric may not always represent a strong link between habitat stability and ecological response. We found a similarly weak link between habitat stability and %EPT, where no clear trend in % EPT is observed across the years at either site. While we did find a trend of %EPT in R-DS1 gradually surpassing %EPT at R-US in the years following the initiation of dam removal, the differences are small relative to within-year variability in the communities.

## Conclusions

The increasing frequency of dam removals [71] provides opportunities to study the linkages between abiotic and biotic responses to disturbance. To investigate these linkages, we monitored changes in characteristics of the channels and benthic macroinvertebrate communities in a BACI study design at two sites in different physiographic settings. In particular, we were interested in decoupling the effects of the elimination of the press disturbance of dams and the introduction of a pulse disturbance with dam decommissioning due to sediment releases. Observations of spatial and temporal patterns in channel features and benthic assemblages indicate that: 1) the presence of the dams constituted a stronger ecological disturbance to the near-downstream reaches on both rivers, as predicted by the serial discontinuity concept, than the pulse disturbance of the dam removal, despite the fact that both rivers passed unregulated flow and sediment during the high flow season; 2) ecological recovery from this press disturbance occurred within the year following the restoration action of dam removal, despite signals of lingering geomorphic disturbance from the sediment released with dam removal, and 3) the analysis of functional traits further confirmed our finding that upstream and downstream sites were ecologically distinct prior to dam removal, but did not add any evidence that the ecological disturbance persisted beyond the period reflected in taxonomically-defined assemblages. These results provide insight into the spatial and temporal extent of the geomorphic and ecological disturbances from dam removal, as well as the trajectories of the recovery from the disturbance of dams and their removal. However, the relatively small sizes of the dams and physiographic settings of the Pacific Northwest is clearly limited in scope and thus results should be confirmed under conditions of larger sediment pulses under a range of physiographic settings.
